# Shared evolutionary footprints suggest mitochondrial oxidative damage underlies multiple complex I losses in fungi

**DOI:** 10.1098/rsob.200362

**Published:** 2021-04-28

**Authors:** Miquel Àngel Schikora-Tamarit, Marina Marcet-Houben, Jozef Nosek, Toni Gabaldón

**Affiliations:** ^1^ Life Sciences Department, Barcelona Supercomputing Centre (BSC-CNS), Jordi Girona, 29, 08034 Barcelona, Spain; ^2^ Institute for Research in Biomedicine (IRB Barcelona), The Barcelona Institute of Science and Technology, Baldiri Reixac, 10, 08028 Barcelona, Spain; ^3^ Department of Biochemistry, Faculty of Natural Sciences, Comenius University in Bratislava, Ilkovičova 6, 842 15 Bratislava, Slovakia; ^4^ Catalan Institution for Research and Advanced Studies (ICREA), Barcelona, Spain

**Keywords:** phylogenomics, complex I, yeast, oxidative phosphorylation, oxidative stress, mitochondria

## Abstract

Oxidative phosphorylation is among the most conserved mitochondrial pathways. However, one of the cornerstones of this pathway, the multi-protein complex NADH : ubiquinone oxidoreductase (complex I) has been lost multiple independent times in diverse eukaryotic lineages. The causes and consequences of these convergent losses remain poorly understood. Here, we used a comparative genomics approach to reconstruct evolutionary paths leading to complex I loss and infer possible evolutionary scenarios. By mining available mitochondrial and nuclear genomes, we identified eight independent events of mitochondrial complex I loss across eukaryotes, of which six occurred in fungal lineages. We focused on three recent loss events that affect closely related fungal species, and inferred genomic changes convergently associated with complex I loss. Based on these results, we predict novel complex I functional partners and relate the loss of complex I with the presence of increased mitochondrial antioxidants, higher fermentative capabilities, duplications of alternative dehydrogenases, loss of alternative oxidases and adaptation to antifungal compounds. To explain these findings, we hypothesize that a combination of previously acquired compensatory mechanisms and exposure to environmental triggers of oxidative stress (such as hypoxia and/or toxic chemicals) induced complex I loss in fungi.

## Graphical abstract

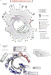

## 1. Introduction

The mitochondrial oxidative phosphorylation (OXPHOS) is a central bioenergetic pathway in eukaryotes [1]. The OXPHOS pathway comprises five membrane-associated multi-protein complexes, of which NADH : ubiquinone oxidoreductase (complex I, CI) is the main entry gate and regulatory hub [2]. CI comprises dozens of subunits encoded in the nuclear and mitochondrial genomes [2]. Besides its central bioenergetic role, CI has been associated with other relevant processes. For instance, in fungi, CI regulates secondary metabolism, virulence and drug sensitivity [3,4], whereas in mammals, CI deficiency can be a driver of tumorigenesis [5–7], and a threat for cell survival [8]. In addition, CI defects are the most frequent source of human mitochondrial disorders, suggesting that it is a fundamental metabolic player [9].

Despite these important roles, several fungal organisms, such as the yeasts *Saccharomyces cerevisiae*, *Candida glabrata* or *Schizosaccharomyces pombe* have lost CI, while retaining the other OXPHOS components [10–12]. This has also been reported in *Plasmodium falciparum* (an apicomplexan parasite causing malaria [13]), *Viscum album* (a parasitic plant [14]) and *Perkinsela* (an endosymbiotic kinetoplastid [15]). The current hypothesis for the loss of CI in yeasts purport these events as convergent adaptations to fermentative lifestyles, where CI is not essential. Two lines of empirical evidence support this hypothesis. First, the finding that duplication of alternative NADH dehydrogenases (NDH2) are associated with CI loss [10], which may be partially redundant with CI function and facilitate a subsequent loss. Second, the lack of alternative oxidases (AOX, often related to respiration) in some CI-devoid organisms has been proposed to reflect adaptation to fermentative or anaerobic lifestyles [10]. However, this idea was postulated from studies of only two fungal clades—*Saccharomycetaceae* and *Schizosaccharomyces*—that had lost CI a long time ago and therefore limited the resolution at which events linked to CI loss could be reconstructed. It is thus generally accepted that the causes and consequences of losing CI remain obscure [10,11]. Clarifying these could lead to a better understanding of the role of CI in multiple cellular processes, and eventually suggest new therapeutic targets for pathogens lacking this ‘essential’ component.

We here exploited available sequence information to discover lineages in which CI was recently lost in the presence of the other OXPHOS components (i.e. independent of complete loss of mitochondrial DNA). We mapped eight such CI losses on the tree of eukaryotes, six of which occurred within fungi. We sequenced and assembled the genomes of four fungal species which, together with available genomes allowed a detailed reconstruction of evolutionary events accompanying three recent CI losses. Our results suggest a link of CI loss with stresses caused by hypoxia and/or antifungal compounds, and reveal additional impacts on metabolism likely related to changes in lifestyle.

## 2. Material and methods

### 2.1. Identifying complex I loss in eukaryotic mitochondrial genomes

We obtained all mitochondrial genomes from the NCBI Nucleotide database (on November 2018), using the search terms ‘complete genome’ and filtering by eukaryotic genomes and mitochondrial DNA. For some species, we found a large number of mitochondrial genome assemblies were available. These could be from re-sequencing projects of the same strain or from different strains. As an example, we found 132 *S. cerevisiae* genomes on 21 January 2021. In the cases where more than 50 assemblies were available, we randomly selected 50 genomes for each species (referenced as taxon IDs). For each genome, we downloaded the gene annotation (if available) and parsed it to identify the annotation of each of the seven CI subunits. We also inferred the presence of the seven CI subunits from *exonerate* (v.2.2)*-*based [16] gene prediction from all conserved mitochondrial CI genes [10], obtained from the KEGG database (release January 2019) (all the fungal proteins that map to the KEGG orthologue clusters K03878, K03879, K03880, K03881, K03882, K03883 and K03884). A subunit was considered absent in a given mitochondrial genome, if it was not annotated and there was no exonerate prediction covering at least 50% of the protein-coding region. We predict that those species that lack at least 5 out of 7 mitochondrial subunits also lost the complex, which is consistent with the bimodal distribution of CI subunits across the mitochondrial genomes (electronic supplementary material, figure S1A,B). Genomes with less than three CDS annotations were considered incomplete and filtered out. We manually curated each of the predictions to discard those coming from incomplete (i.e. genomes that are annotated as ‘complete genome’ but that are related to a paper which states that only a part of the genome was sequenced) or misannotated genomes (i.e. genomes that have annotations for a given subunit, like ‘protein with homology to NADH : ubiquinone dehydrogenase subunit 1’ instead of ‘NAD1’, would not be considered as CI deficient). The definition of ‘independent loss’ was based on identifying nodes in the phylogenetic species tree (inferred from taxonomic information [17]) monophyletic for CI absence or presence. We consider as ‘ancient’ loss one that occurred on species of multiple genera, whereas ‘recent’ losses are those found in species that have closely related taxa (within the same genus) with CI. Electronic supplementary material, table S1 includes all these results.

### 2.2. Genome sequencing and assembly of *Wickerhamomyces* and *Ogataea* species

We sequenced the genomes of strains that lacked CI subunits in the mitochondrial genome of the following species: *Wickerhamomyces pijperi* CBS2887, *Wickerhamomyces mucosus* CBS6341, *Ogataea philodendri* CBS6075 and *Ogataea thermophila* NCAIM Y.01608. The CBS strains were from ‘Centraalbureau voor Schimmelcultures' (now Westerdijk Fungal Biodiversity Institute, Utrecht, the Netherlands). The *O. thermophila* strain was provided by Dr Gábor Péter (National Collection of Agricultural and Industrial Microorganisms, Budapest, Hungary [18]). We performed the quality control of the reads with *fastqc* (v.11.8) http://www.bioinformatics.babraham.ac.uk/projects/fastqc, and the trimming with *trimmomatic* (v.0.38) [19]*.* We used *SPAdes* (v.3.9) [20] for *de novo* genome assembly, and *redundans* (v.0.14) [21] or *dipSPAdes* (v.3.9) [22] for reduction of heterozygosity. We compared the results of the three assemblies (based on *SPAdes, dipSpades and SPAdes followed by redundans*), and kept the best one in terms of largest N50 and minimum number of scaffolds. In all cases, the *redundans* reduction yielded the best assemblies. The information of each of the assemblies can be found in electronic supplementary material, table S2. The raw reads have been deposited in SRA under BioProject PRJNA663430. The genomes can be found in GenBank through the same BioProject.

### 2.3. Phylogenomic analyses

To reconstruct the phylomes of species that recently lost CI we implemented a previously described pipeline [23], which generates a phylogenetic tree for the homologues (in a set of ‘target’ proteomes) of each of the proteins of a given ‘seed’ proteome. We generated phylomes for six seed proteomes (predicted with *Augustus* from the corresponding genomes, including *O. philodendri, O. thermophila, W. pijperi, W. mucosus, S. bacillaris, S. bombicola*) with 23 ‘target’ proteomes, which include fungal species closely related to each of the species with a recent CI loss event and two outgroups (electronic supplementary material, figure S2A). For the analyses of genes lost and duplicated, we discarded orthologous groups that have similar evolution in CI− species and some of their close CI+ relatives (in the sister group), as these represent changes that are not specifically associated with CI loss.

To analyse relevant fungal gene families (electronic supplementary material, figure S3) we generated one phylogenetic tree for each gene within the several categories suggested in the analysis of recent CI loss. For each ‘seed’ gene (1127 in total), we inferred orthologue clusters in a set of proteomes (from a selection of 98 species covering all the nodes monophyletic for CI−/CI+ (electronic supplementary material, figure S1F)). For each cluster, at each ancestral node monophyletic for CI+/CI−, we calculated the median number of orthologues for each species. To identify relevant clusters (convergently associated with CI loss) we evaluated the statistical significance (using a Kolmogorov–Smirnov test, *p* < 0.05) of the difference between the median number of orthologues in the CI+ and CI− nodes.

### 2.4. Functional annotations and enrichment analysis

We used *InterProScan* (v.5.32) [24] to annotate *Pfam* domains and *Prosite profiles/patterns* of the relevant genes. We ran BLASTkoala (on January 2019) [25] to annotate the KEGG orthologues and pathways related to each gene within the CI− species and those in their sister group (as identified in the phylome reconstruction (electronic supplementary material, figure S2A)).

We performed gene ontology (GO) enrichment analyses with the *goatools* package (v.0.8) [26], using the false discovery rate (FDR) for multiple test comparison correction. We identified as GO terms convergently associated with recent CI loss those that were significantly enriched (without multiple testing correction) in all CI losses. Avoiding multiple testing correction was necessary here to decrease false negatives in categories with few genes, such as those in the intersection between duplication and positive selection (PS) (table 1). In addition, we consider that only relying on significant terms in all three CI losses is already a (biological) correction of the eventual false positives derived from using raw *p*-values. See electronic supplementary material for more details.

**Table 1.** Enriched GO terms in all species with recent complex I loss. All such GO terms are related to transmembrane transport (TM transport) and oxidation–reduction processes (OR process), and can only be found in genes under loss, duplication and duplication and positive selection. ‘X’ reflects that the GO enrichment analysis yielded a *p* < 0.05 in all species with recent CI loss, while ‘XX’ indicates that the *p-*value after FDR correction was also below 0.05. ‘BP’, ‘CC’ and ‘MF’ stand for biological process, cellular component and molecular function, respectively.

GO term	duplicated	loss	dup. and +sel.	group
BP: establishment of localization	X		X	TM transport
BP: localization	X		X	TM transport
BP: oxidation–reduction process	X	X		OR process
BP: transmembrane transport	XX	XX	X	TM transport
BP: transport	X		X	TM transport
CC: integral component of membrane	XX	XX	X	TM transport
CC: intrinsic component of membrane	XX	XX	X	TM transport
CC: membrane part	XX	X	X	TM transport
MF: ATPase activity, coupled to transmembrane movement of substances			X	TM transport
MF: P-P-bond-hydrolysis-driven transmembrane transporter activity			X	TM transport
MF: active transmembrane transporter activity	X			TM transport
MF: flavin adenine dinucleotide binding		X		OR process
MF: oxidoreductase activity	X	X		OR process
MF: primary active transmembrane transporter activity			X	TM transport
MF: transition metal ion binding	X			OR process
MF: zinc ion binding	X			OR process

### 2.5. Positive selection analysis

We interrogated PS on genes in species with recent CI loss (CI− genes) as compared to the orthologues of the close CI+ species (defined as those in the sister groups and immediate outgroups in the species tree (electronic supplementary material, figure S2A)). For each CI− gene, we generated the codons' phylogenetic tree together with the close orthologues (using *MAFFT, pal2nal* (v.14) [27] **and* iqtree* with automatic model selection) and ran *codeml* (v.4.8) [28] to test for differences between the relaxation and PS brach-site models [29] at the CI− gene. We defined as ‘positively selected’ CI− genes those that passed the test with a *p*-value below 0.05 after FDR correction.

We also interrogated the statistical significance of the observation that eight gene families are under PS in all three species with recent CI loss. We modelled the null hypothesis (the observed overlap is explained by chance) by generating 10 000 randomly assigned PS calls to genes (keeping the total number of calls per species), together with the calculation of the resulting number of overlapping families between species. From this distribution, we inferred a probability (*p*-value) to find at least eight overlapping families under PS.

### 2.6. Subcellular localization and mitochondrial retargeting prediction

We used *deeploc* (v.1.0), a deep learning-based predictor of subcellular localization [30], which inputs protein sequences and outputs a probability for several subcellular localizations. The predicted compartments are ‘mitochondrion’, ‘cytoplasm’, ‘nucleus’, ‘peroxisome’, ‘lysosome/vacuole’, ‘golgi apparatus’, ‘endoplasmic reticulum’, ‘plasma membrane’ and ‘extracellular’. In addition, *deeploc* also outputs a probability of ‘membrane’ localization. Given that protein localization can be shared across compartments in yeast [31], we reasoned that the inference of retargeting cannot be simplified to identifying pairs of orthologues with different ‘main locations’ (as predicted from the compartment with the highest predicted probability). We hypothesized that, for a given compartment, the quantitative difference in probabilities between orthologues may be predictive of retargeting (electronic supplementary material, figure S2C). To test this we used an available dataset of subcellular localization measurements in yeast [31], which allowed us to identify pairs of paralogues (inferred from the generated phylome; electronic supplementary material, figure S2A) that are (or not) under mitochondrial retargeting (MR). For each pair of paralogues, we calculated the differences in predicted mitochondrial probability (with *deeploc*) and defined as retargeted those pairs surpassing a given threshold. We found that our method was indeed accurate to predict MR between homologous sequences (electronic supplementary material, figure S2D,E), indicating that it can be useful for inferring genes that acquired mitochondrial location in CI− species. We identified as ‘mitochondrially retargeted’ CI− genes those that have a mitochondrial probability greater than 0.2 as compared to any of the sister CI+ orthologues. This is the threshold that maximizes sensitivity with high precision (0.71) according to our yeast benchmarking analysis (electronic supplementary material, figure S2E). This model considers the possibility of multi-localization, so that retargeting may just involve a fraction of all proteins.

### 2.7. Inferring the genomic changes associated with CI loss on KEGG pathways

In order to visualize the effect of evolutionary changes (gene loss (L), duplication (D), PS and MR) on pathways we built a pipeline that draws KEGG pathways (obtained through *BLASTkoala* and the KGML package of Biopython (v.1.72) [32]) showing each of the genes (represented according to the evolutionary alterations they experienced) that map to the corresponding enzymes. For each enzyme, the genes are represented (for each species) with colours indicating the level of similarity, as described in [25]).

From this enzyme-to-gene remapping, we ranked all KEGG pathways according to the convergent evolutionary changes affecting them in CI loss (electronic supplementary material, figure S4A). For a given species, the score of each pathway was calculated as the sum of the scores of its enzymes. The score of each enzyme was the sum of two co-occurrence scores (assigned if any of the genes were under PS, MR and/or D in two (*p1*) or three (*p2*) of the CI− species), a uniqueness score (*p3*, assigned if the enzyme was lost or gained with CI loss, as compared to close CI+ species) and the global gene score. The latter was calculated as the sum of the individual scores of genes mapping to the enzyme, which were positive for genes belonging to the PS, MR, D (medium score, *p4*) or loss (lower score, the *p5*) categories, and negative (*sixth parameter*) otherwise. To prioritize high-confidence predictions, each individual gene score was multiplied by 10 or 1 for high and low identity gene-KEGG orthologue (as classified by *BLASTkoala*) mappings, respectively. The absence of genes mapping to a given enzyme in any of the species would add a negative score to the pathway (equal to *p6*). We could not design any meaningful calculation of these six parameters (*p1*–*p6*), so that we scored the pathways with 729 combinations where each parameter would range between:

$$\begin{array}{rl} & p1\in (\;p3+10,\;100);p2\in (\;p1+20,\;200);p3\in (\;p4+5,\;50);\\ & \;p4\in (\;p5+1,\;10);p5\in (0.5,\;5);p6\in (\;p5+1,\;10) \end{array}$$

For each set of parameters (and for each species), we calculated the *z*-score of all KEGG pathway scores, so that we calculate the ‘final’ score as the mean of all the *z*-scores. We then inferred the top 10 affected pathways as those with the highest mean (across species) final scores. We note that this model slightly prioritizes gene innovation (D, PS, MR) over the single-orthologue loss, under the assumption that losing one gene copy probably reflects diminished enzymatic activity rather than innovation.

## 3. Results

### 3.1. A cartography of complex I loss across the eukaryotic tree of life

**Figure 1.**
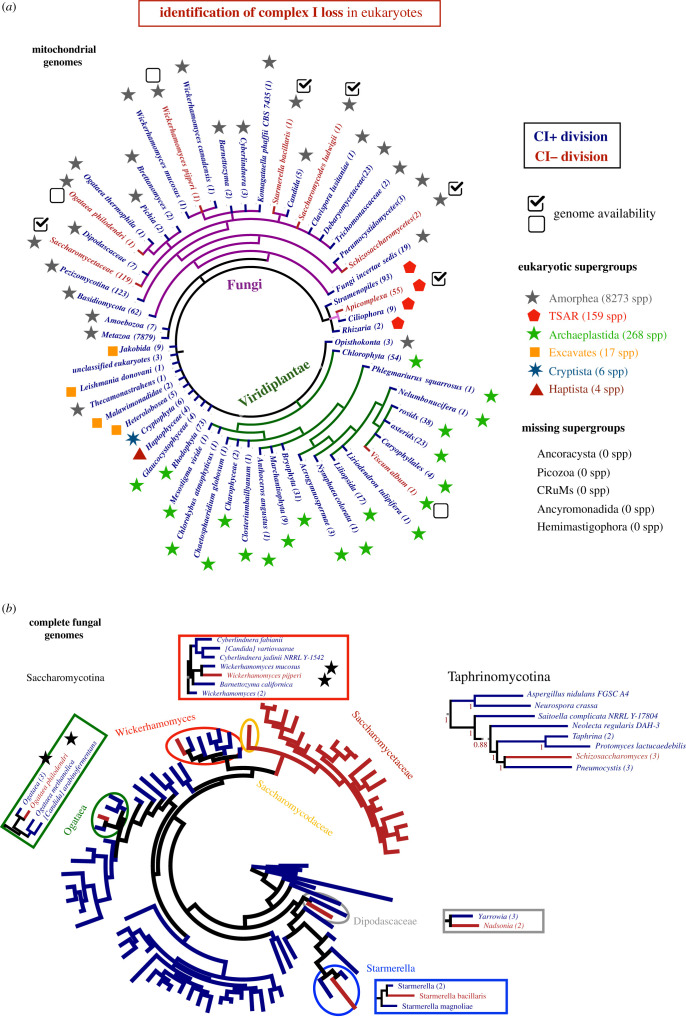
Identification of complex I loss in eukaryotic genomes. (*a*) Loss of complex I (CI) from the oxidative phosphorylation (OXPHOS) system in eukaryotes, as inferred from the analysis of mitochondrial genomes. CI was lost in at least eight eukaryotic divisions (in red), within Viridiplantae (green), Alveolata (pink) and Fungi (purple). The species tree shown is based on NCBI taxonomy. The boxes show the availability of complete genomes within each of the divisions at the time of this study. Each tip represents a division of the species tree that is monophyletic for the presence (blue) or absence (red) of CI. The number of species included in each division is indicated in parenthesis. Symbols indicate the eukaryotic supergroup to which each division belongs (based on [33]), which reflects the coverage of eukaryotic diversity in our analysis. Note that we could not find mitochondrial genomes in the database for five of these supergroups at the time of this study. (*b*) Whole genome-based phylogenetic reconstruction of a representative set of species in Saccharomycotina (left) and Taphrinomycotina (right) suggest six independent CI loss events in fungi. For simplicity, the tips of the trees do not always correspond to species, but collapsed groups of species from the same genus that are monophyletic for the presence or absence of CI. The branch lengths correspond to the last common ancestor of all the species in each division. The insets clarify the specific topology (showing all species/genus names) of the loss events, and include species for which the genome was sequenced in this study (marked with stars). The complete Saccharomycotina tree with all species/genus names and branch supports can be found in electronic supplementary material, figure S1F. The numbers shown on the Taphrinomycotina tree branches indicate bootstrap support, 1 being the maximal support.

We exploited the large availability of genomic sequences in public databases to derive an updated cartography of CI loss. Nuclear and mitochondrial subunits of CI are always co-lost [10,12], and thus the absence of mitochondrially encoded CI subunits is a good proxy for complete CI loss. We combined genome mining with homology-based tools to screen 51 350 mitochondrial genomes from 10 666 eukaryotic organisms (see Material and methods; figure 1*a*; electronic supplementary material, figure S1A,B and table S1). We integrated this information with species taxonomic classification [17], which suggests that there have been at least eight independent losses of CI distributed across fungi, plants and protists. Most losses (six out of eight) comprise fungal taxa, which include whole families (*Saccharomycetaceae* and *Schizosaccharomycetaceae*) and four individual species (*Wickerhamomyces pijperi*, *Starmerella bacillaris*, *Ogataea philodendri* and *Saccharomycodes ludwigii*). We also detected the previously reported CI loss in the plant *V. album* [14,34] and in *Apicomplexa,* which include *Plasmodium* species [35,36] (figure 1*a*). We could not find the previously reported CI loss in *Perkinsela* [15], due to the absence of this mitochondrial genome in the screened database. Importantly, the high number of these events in fungi and their amenability for genome sequencing indicate that they represent ideal organisms to study genomic changes associated with CI loss. Remarkably, we identified two fungal genera showing recent CI losses (those in the budding yeasts *W. pijperi* and *O. philodendri*, which have close relatives with CI) for which full genomes were not available at the time of this study. Comparative studies that correlate a given trait (i.e. CI loss) with genomic changes are more robust between closely related organisms [37]. We sequenced and assembled the complete genomes for these and closely related species (*W. mucosus* and *O. thermophila*) (electronic supplementary material, table S2), and combined them with the available complete fungal genomes totalling 1925 fungal species. Of note, some of the species without CI also had no mitochondrial genomes in the database. These cases do not allow to differentiate CI loss from loss of the entire mitochondrial genome, and were not considered in further analyses.

To provide a phylogenetic framework to our analysis, we generated a whole genome*-*based species tree for a selection of 144 organisms in Saccharomycotina and Taphrinomycotina (ascomycete subphyla, see electronic supplementary material), using two Pezyzomycotina genomes as outgroups. We screened these genomes for CI loss through homology-based gene prediction (electronic supplementary material, figure S1C). Mapping CI loss on the tree and using a parsimony approach, we inferred one event in Taphrinomycotina and five independent events within Saccharomycotina*,* including (i) *W. pijperi*, (ii) *O. philodendri,* (iii) *S. bacillaris*, (iv) the common ancestor of *Saccharomycodaceae* and *Saccharomycetaceae*, and (v) the genus *Nadsonia* (figure 1*b*). Importantly, we confirm using whole genomes that CI was recently lost in the genera *Ogataea, Wickerhamomyces* and *Starmerella*, previously predicted from mitochondrial sequences [38,39]. Taken together, our results suggest that CI loss is not uncommon in fungi, particularly in yeasts.

### 3.2. Identifying convergent genomic changes associated with the recent loss of complex I

The discovery of multiple recent independent losses of CI in fungi opened, for the first time, the possibility to identify convergent processes associated with this event. Our assumption is that finding genomic changes recurrently associated with CI loss may be useful to predict the evolutionary causes of the loss, and/or infer its immediate consequences. To this end, we first reconstructed the phylomes (the set of phylogenetic trees for all genes in a genome) for species that recently lost CI (CI− hereafter) and their close, CI-bearing relatives (CI+). This allowed us to establish phylogeny-based orthology and paralogy relationships among genes as well as infer events of gene duplication (D) and loss (L) (electronic supplementary material, figure S2A). We also predicted genes under positive selection (PS) in CI− species, which may include those with neofunctionalization or subfunctionalization related to CI loss (electronic supplementary material, figure S2B). In addition, given the mitochondrial localization of CI, we reasoned that mitochondrial retargeting (MR) [40] of certain proteins may compensate and/or promote CI loss. We thus used a method that allows the accurate sequence-based prediction of MR events (electronic supplementary material, figure S2C,E) to identify such events in lineages that underwent CI loss (see electronic supplementary material).

**Figure 2.**
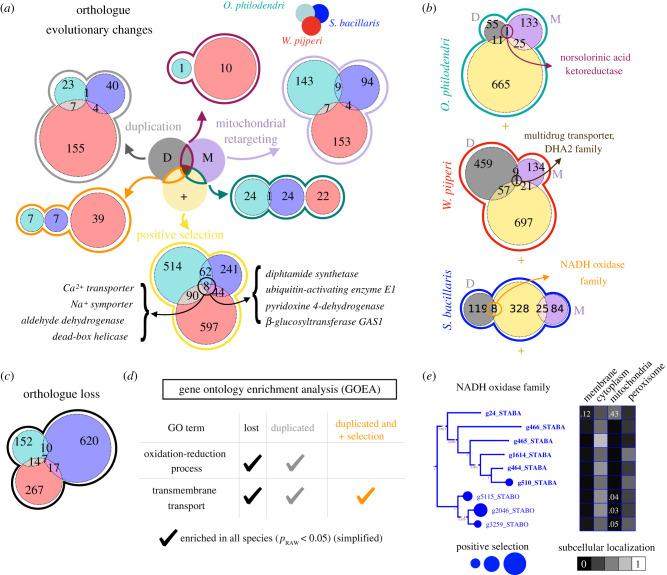
Finding common genomic footprints of recent complex I loss. We reconstructed the phylomes for the three species that underwent recent CI loss (*O. philodendri, S. bacillaris* and *W. pijperi*) to find orthologous gene families that underwent convergent evolutionary changes. (*a*) Hundreds of orthologous families are under duplication (D, grey), positive selection (+, yellow), mitochondrial retargeting (M, purple) in each of the species (depicted as green, blue, red colours). The Venn diagrams show the number of families found in each species under each type of evolutionary change (D, M, +). For example, there is only 1 orthologous family with duplications in both *O. philodendri* and *S. bacillaris.* Importantly, eight families have genes under positive selection in all species, whose function is shown as a simplification of the InterProScan and KEGG annotations. Phylogenetic trees of each family can be found in electronic supplementary material, figure S5. We calculated the probability of having at least eight of such overlapping clusters under random assignation of positive selection to genes (keeping the total number of calls constant) which yielded a *p* < 0.0024. (*b*) The same as (*a*), but sowing the overlap across different evolutionary changes in the same species. For example, there is only one family (including proteins annotated as norsolorinic acid ketoreductases) with genes under both duplication (D) and mitochondrial retargeting (M) in *O. philodendri*. We note that there is one gene under M, D and + in *W. pijperi* annotated as a DHA2 multidrug transporter. In addition, we find one NADH oxidase under D and + in *S. bacillaris.* Further annotations of these families are provided in electronic supplementary material, table S3. (*c*) Several families are co-lost with CI, 7 of them in all species, depicted as in (*a*). Phylogenetic trees of each family can be found in electronic supplementary material, figure S6. (*d*) We evaluated whether some GO categories were enriched in the gene sets shown in (*b,c*) for all species under recent CI loss. This revealed that oxidation–reduction process and transmembrane transport are enriched in genes under loss, duplication and/or positive selection. Table 1 includes more details. (*e*) The phylogenetic tree for a NADH oxidase family in *S. bacillaris* (STABA) and *S. bombicola* (STABO) (which all have such an InterPro annotation) under both duplication and positive selection (outlined in (*b*)). This panel also shows information related to the evolutionary changes in each gene. The circles represent the −log_10_(p) of the positive selection test for each gene. The genes in bold correspond to species without CI. The grid represents the predicted score of subcellular localization in membrane, cytosol, mitochondria and peroxisome, respectively. Note that this score can be split among several compartments because some proteins could have several locations. Shown are only those compartments with a score greater than 0.1 in at least one gene. STABA stands for *S. bacillaris* and STABO for *S. bombicola*.

All in all, we scanned for gene families experiencing L, D, PS and/or MR in the CI− lineages. We found 1976 families with at least one such event in any of the CI− lineages. Most remarkably, we found eight gene families with PS in all CI− species, which is far from random expectation (*p* = 0.0023). This set includes the orthologues of *S. cerevisiae* PMR1, DPH6, YPR127 W (putative pyridoxine-4 dehydrogenase), ALD4, ALD6, ALD5, DED1, DBP1, GAS1, UBA3, and a paralogue of *S. cerevisiae* DUR3 (annotated as ‘sodium symporter’) (figure 2*a*; electronic supplementary material, figure S5). Similarly, we found seven gene families that were independently lost in all CI- species, suggesting strong functional association with CI [41]. Three of these have homology to CI assembly factors 3, 6 and 7, respectively; and three of the others have predicted mitochondrial membrane location (the same compartment as CI), which further supports a possible role as CI-associated factors (figure 2*c*; electronic supplementary material, figure S6). We thus predict that the protein products of these gene families are functionally related to CI.

We reasoned that the genomic changes related to CI loss may not be reflected in convergent gene family alterations, but in common cellular functions exerted by different families in each species. To test this we identified GO terms enriched in all CI− species for the genes belonging to each of the L, D, PS, MR categories and the ones in all possible intersections. Oxidation–reduction process is enriched among D and L genes, suggesting a diversification in the oxidative metabolism upon CI loss. Several terms related to transmembrane transport are enriched in D or D + PS genes, suggesting that CI loss might have been associated with exposure to different compounds, which required novel transporters (table 1, figure 2*b*,*d*; electronic supplementary material, tables S3 and S4). In addition, we manually explored the gene trees and annotations of all genes in the intersections between D, PS, MR to identify single genes potentially associated with CI loss. We found a NADH oxidase under extensive duplication in *S. bacillaris*, with one of the copies retargeted to mitochondria (figure 2*e*) which may perform a biochemical function analogous to CI that compensates its loss. All together, our data suggest that several biological functions are convergently altered upon (or as a result of) CI loss, which is consistent with its roles beyond energy production [3,42].

### 3.3. Inferring the specific pathway changes related to recent complex I loss

To more precisely investigate the metabolic context of the above mentioned altered genes we developed a pipeline that visualizes genes, evolutionary processes and metabolic pathways for CI− species (electronic supplementary material, figure S4A, see electronic supplementary material). This tool helped us to explore specific hypotheses about the causes and consequences of CI loss. As an example, we found that all the enzymes related to transforming pyruvate to ethanol (alcoholic fermentation) experienced PS and/or D in all CI− species (electronic supplementary material, figure S4A), suggesting that CI loss in these species is associated with a shift towards fermentative metabolism (as previously proposed [10]). However, we noted that such co-duplication and co-positive selection events may be frequent in yeasts (up to 40% depending on how we infer them, electronic supplementary material, figure S7), suggesting that changes in fermentation may be recurrent in fungal evolution, and not specific to CI loss (as previously proposed [43]). We generated such representations for all KEGG pathways with any affected genes, which we provide as supplementary material (electronic supplementary material, DataSet S1).

We next used a statistical framework to score all pathways from convergent evolutionary alterations (D, PS, MR, L) (electronic supplementary material, figure S4A), and identified the most affected cellular functions. Each species yielded a different set of top-scoring pathways (electronic supplementary material, table S5), which may reflect that metabolism in different CI− organisms has taken somewhat diverse evolutionary paths. However, three pathways were consistently among the ten top-scoring pathways. These include retinol metabolism, chloroalkane–chloroalkene degradation and cytP450-based drug metabolism (electronic supplementary material, figure S4B). We note that annotations do not always equate to confirmed biochemical function, indicating that these pathways could be slightly different in CI− organisms. This motivated us to further dissect the source of these high scores by visualizing the convergently modified enzymes of these pathways (electronic supplementary material, figure S4C). These include an alcohol dehydrogenase (ADH EC 1.1.1.1) in the three pathways, an aldehyde dehydrogenase (AdDH EC 1.2.1.3) in chloroalkane degradation, and a glutathione-*S*-transferase (GST EC 2.5.1.18) in drug metabolism. An isolated analysis of the three pathways suggests that CI loss is predominantly associated with a change in retinol metabolism, and novel chloroallyl alcohol and aldophosphamide degradation (which are toxic halogenated compounds [44,45]). However, ADH and AdDH are also related to ethanolic fermentation (electronic supplementary material, figure S4A), which is more likely to be associated with CI loss [10]. In addition, retinol and GST enzymes have been described as mechanisms protective of mitochondrial oxidative stress [46,47], which may be related to the role of CI in the generation of reactive oxygen species (ROS) [48,49]. In fact, there are putatively mitochondrial GST copies (*p*(mitochondrial) > 0.1) duplicated in two of the CI− species (*W. pijperi* and *S. bacillaris*). The evolutionary changes in these enzymes may reflect that CI loss is associated with an increase in mitochondrial antioxidant systems, consistent with the observed duplication and loss of oxidoreductases (figure 2*d*). However, the implication of these enzymes in multiple pathways hampers a more precise functional interpretation.

Finally, we focused on pathways and KEGG orthologues with genes in the intersections between PS, D and MR in at least two of the CI− species. A yeast amino acid transporter (ko:K16261) was the only such KEGG orthologous family (which comprises 16 different orthologous gene families according to our more fine-grained phylome analysis) (electronic supplementary material, figure S4D), which is a source of the D–PS transporters (table 1). In addition, we found two pathways with these signs of innovation: ABC transporters and glycerolipid metabolism (electronic supplementary material, figure S4E). Among the ABC transporters, the orthologues of *S. cerevisiae* PDR5 and SNQ2 (multidrug resistance transmembrane transporters) are under PS + D in *S. bacillaris* and *W. pijperi*, respectively. Additionally, SNQ2 orthologue shows PS in *O. philodendri* (electronic supplementary material, figure S8). This may suggest toxic compound exposure associated with CI loss. This notion is consistent with the convergent enrichment of active transporters in D + PS genes (table 1). Within glycerolipid metabolism, we found several enzymes PS + D or MR + PS (in all species) involved in glycerol metabolism and fatty acid degradation (electronic supplementary material, figure S4E). This suggests that CI loss induced a drastic change in these processes, which actually depend on mitochondrial respiration [50].

### 3.4. Recent complex I loss and the evolution of antioxidant systems

It has been observed that reoxygenation after periods of hypoxia (often faced by facultative anaerobes) trigger the formation of ROS in CI [11], which we hypothesize to partially explain the advantage of its loss in such lifestyles [10]. Our analysis of recent CI loss suggests that it is associated with novel antioxidants (electronic supplementary material, figure S4C) but also to adaptation to antifungal compounds (electronic supplementary material, figure S4E). This second process had never been considered as a possible driving force for CI loss. However, some antifungal compounds are known to generate ROS derived from mitochondrial respiration [51–53]. In this regard, CI deletion has been shown to modulate resistance to azole drugs [3,42]. Given that CI is one of the main sites of mitochondrial ROS generation [48,49], we hypothesize that CI loss might have resulted as an adaptation (together with the PS and/or D in PDR5 and SNQ2 orthologues) to the presence of toxic compounds eliciting mitochondrial ROS. Many such ROS-generating antifungal compounds are natural products, such as phenazines [52], amphotericin B, caspofungin [52,54] or azoles [55]. We thus reasoned that the presence of ROS-generating toxic compounds in the environment could have promoted CI loss in these lineages, probably in combination to adaptation to hypoxic environments. This would explain the convergent evolutionary forces acting in drug efflux transporters.

**Figure 3.**
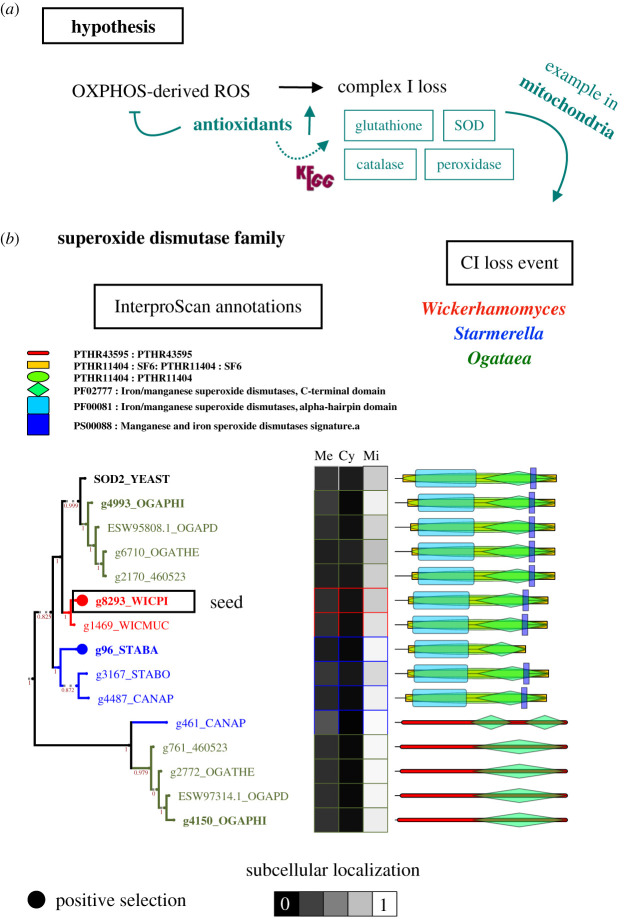
Inferring the evolution of antioxidant mechanisms on species with recent complex I loss. (*a*) We hypothesize that increased antioxidant mechanisms may buffer mitochondrial ROS that induce and/or result from CI loss. We analysed genes under duplication (D), positive selection (PS) or mitochondrial retargeting (MR) in glutathione-related proteins, catalases, peroxidases or superoxide dismutases (SOD). The trees of these genes can be found in electronic supplementary material, figures S9 and S10. (*b*) An example gene family (with SOD annotations) suggests convergent MR, D and/or PS of antioxidant systems (mitochondrial SOD). The tree shows evolutionary and functional information for each gene (similarly to figure 2*e*) to aid interpretation. The circles represent the −log_10_(p) of the positive selection test. The boxes represent InterProScan annotations, and the grid the prediction score of subcellular localization (membrane (Me), cytosol (Cy), mitochondria (Mi), respectively). Shown are only those compartments with a score greater than 0.1 in at least one gene. The genes in bold correspond to species without CI. The colour indicates the genus of each gene (note that *Wickerhamomyces, Ogataea* and *Starmerella* are the genus with CI loss). The outlined gene is the seed of the tree. STABA stands for *S. bacillaris*, STABO for *S. bombicola,* CANAP for *Candida apicola,* OGAPHI for *O. philodendri,* OGATHE for *O. thermophila,* OGAPD for *O. parapolymorpha,* 460523 for *O. polymorpha,* WICPI for *W. pijperi,* and WICMUC for *W. mucosus* and YEAST for *S. cerevisiae*.

We consider that one way to investigate this is to infer the evolution of known mitochondrial antioxidant systems in CI− species, which may reflect synergistic adaptation to such oxidative stress with CI loss. We explored the trees of all genes in D, PD, MR mapping to any of the KEGG orthologues related to glutathione, superoxide dismutase (SOD), catalase or peroxidase (electronic supplementary material, table S6, and figures S9 and S10); looking for any sign of convergent mitochondrial innovation in CI− species. The orthologues of *S. cerevisiae* SOD2 (mitochondrial SOD) are under PS in *W. pijperi* and *S. bacillaris*, and *Ogataea* species have two copies (figure 3*b*). In addition, the orthologous families related to the GST2.5.1.18 are under PS, D and/or MR in both *W. pijperi* and *S. bacillaris*; and the *Ogataea* clade has one of the copies under MR (electronic supplementary material, figure S9). Taken together, our results indicate that CI loss may be associated with novel mitochondrial antioxidant systems. At least in *O. philodendri*, this happened before CI loss (the order of events cannot be resolved for *W. pijperi* and *S. bacillaris*), suggesting that these lineages had already an increased antioxidant activity that probably allowed CI loss by synergistic ROS detoxification.

### 3.5. Comparing signatures of recent and ancient complex I loss

**Figure 4.**
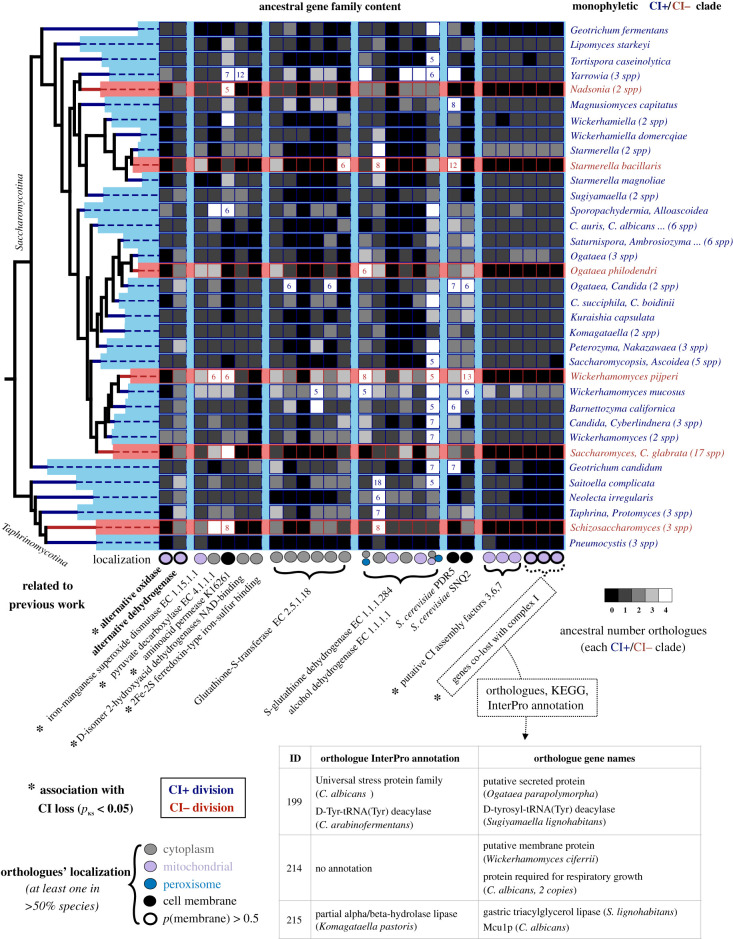
Study of particularly relevant protein families in all CI losses. We inferred the number of orthologues from several gene families potentially related to CI loss (see material and methods, figure 2) in a selection of Saccharomycotina and Taphrinomycotina species. The complete tree with the number of orthologues in each species can be found in electronic supplementary material, figure S3. In order to show the relationship between ancestral CI state and gene content, we here show a collapsed tree where each tip represents a single species or a group of species monophyletic for CI+ (blue) or CI− (red) species. The name of each tip indicates some of the genera found in each division, with the number of included sequenced species in parenthesis. The grey scale represents the predicted ancestral number of orthologues for each gene family in the last common ancestor of these species. This number is inferred as the median number of orthologues across extant species. The families are named after the KEGG and InterProScan annotations, marked with an asterisk when orthologue copy number is significantly associated with CI loss (KS test *p-*value < 0.05). We also predicted the main subcellular localization(s) for each family (circular dots) as those of more than half of the species. The annotation and name of the orthologues (if any) of the genes co-lost with CI that do not map to KEGG CI assembly factors (right-lower corner, figure 2*c*; electronic supplementary material, figure S6C) are also shown.

We next considered whether these emerging hypotheses about recent CI loss could also explain ancient loss events, which includes species like *S. pombe,* and *S. cerevisiae* (electronic supplementary material, figure S1F). To this end, we screened for gene loss and duplication events in the orthologous groups related to recent CI loss (figures 2–4), including a broader taxonomic sampling to cover ancient CI loss. For each family, we approximated the main subcellular localization(s) as the compartment(s) predicted for at least 50% of the species' orthologues.

First, we explored two families considered to be associated with CI loss: AOX and NDH2 [10,11]. We found that AOX loss is always related to CI loss (preceding it in 4/6 events), whereas NDH2 duplication is associated with CI loss (preceding 3/6 events) in all but *S. bacillaris*. This suggests that the ancestral species that lost CI already lacked AOX and had increased alternative NDH2 activity (which could have compensated loss of CI function, as previously described [10,11,56]). In *S. bacillaris*, the increase in mitochondrial NDH2 activity may be mediated through the expanded, mitochondrially retargeted, NADH oxidase family (figure 2*e*).

We next focused on associations between copy number and CI loss, which yielded twelve orthologous clusters (figure 4). These include the same six families always co-lost with CI (electronic supplementary material, figure S6). Three of them have annotations as CI assembly factors 3,6,7 (electronic supplementary material, figure S6A–C) and mitochondrial localization. The other three are probably mitochondrial membrane proteins, and may include a D-Tyr-tRNA(Tyr) deacylase (cluster 199), a protein required for respiratory growth (cluster 214) and a protein whose orthologues are lipases and Mcu1p (required for non-fermentable growth [57]) (cluster 215). We propose that clusters 214 and 215 include uncharacterized proteins necessary for CI function, which is consistent with the co-loss, the predicted compartment (the same as CI, which may be related to physical interaction) and their essentiality for respiration in *C. albicans* orthologues (figure 4)*.* Interestingly, these six families have been also lost in *Brettanomyces bruxellensis* and *Geotrichum fermentans*, both used in industrial fermentation processes [58–60], which suggests that they may rely less on aerobic respiration and can lose CI-related factors. Additionally, the orthologues of the mitochondrial SOD (figure 3*b*) were duplicated in 4/6 CI loss events (probably preceding loss of *S. bacillaris* and *W. pijperi*, and maybe *O. philodendri*; figure 4; electronic supplementary material, figure S3), suggesting that CI− species had an ancestral higher ability to detoxify mitochondrial ROS before CI loss. Furthermore, the pyruvate decarboxylase enzyme (implicated in ethanolic fermentation; figure 3*a*) was duplicated in 4/6 CI losses, supporting the idea that most of these species increased their glycolytic metabolism upon CI loss (figure 4; electronic supplementary material, figure S3). In addition, a D-isomer 2-hydroxyacid dehydrogenase with NAD-binding and a FAD-binding ferredoxin-like protein were lost with CI in 4/6 and 6/6 cases (figure 4), consistent with a diversification in oxidoreductive metabolism upon CI loss. Finally, an amino acid permease (also mentioned in electronic supplementary material, figure S4D) was duplicated in 4/6 cases (figure 4), which may relate to environmental changes driving CI loss.

We explored families related to ADH, GST and drug transport (electronic supplementary material, figure S4C,E) to test the previously mentioned association with fermentation and antioxidant mechanisms. Several families of GSTs were duplicated in all recent CI loss events (preceding the one in *W. pijperi*), which was not true for ancient losses. For ADH, we observed a cytosolic family duplicated in 5/6 CI loss events, likely preceding all recent CI losses. Finally, we observed increased numbers of multidrug transporters (PDR5, SNQ2) in all recent CI loss as compared to close species (figure 4; electronic supplementary material, figure S3).

In summary, the only families that are expanded/contracted in (or preceding) almost all CI loss events (at least 5/6) are the putative CI assembly factors and AOX/NDH2. This may be due to long evolutionary time having blurred the genomic determinants of CI loss in *Saccharomycetaceae* and *Nadsonia.* Finally, we note that broader taxon sampling has allowed us to differentiate gene loss and duplication events happening before (such as AOX loss) or together with CI loss, and suggest that most lineages that lost CI already had expanded alternative NADH hydrogenases and reduced AOX. Importantly, this analysis revealed that convergent alterations in several families preceded CI loss, indicating that they are related to the causes or predisposing factors. Conversely, it is difficult to distinguish causes from consequences for other convergent changes, such as the alteration of fermentative enzymes.

## 4. Discussion

The recurrent loss of CI represents one of the most puzzling observations of metabolic evolution [12]. By studying recent loss events in fungi we have been able to gain insight into the evolutionary routes that made it possible, predicting novel features about CI biology and evolution. First, we have identified three gene families that are recurrently co-lost with CI and likely in the same cellular compartment. We propose these as CI-related factors (two of them necessary for respiration in *C. albicans*), which may represent undescribed CI subunits or assembly factors. Second, our results suggest that the fungal clades that include species with CI loss possess several genomic singularities that may have facilitated CI loss. These include duplication and/or PS of a GST, the mitochondrial SOD, the alternative NDH2, enzymes related to ethanolic fermentation, and the loss of AOX. CI− species have also specific signatures that may reflect evolutionary processes following (or concomitant with) CI loss, including adaptation to antifungal compounds and expansion of fermentative enzymes.

**Figure 5.**
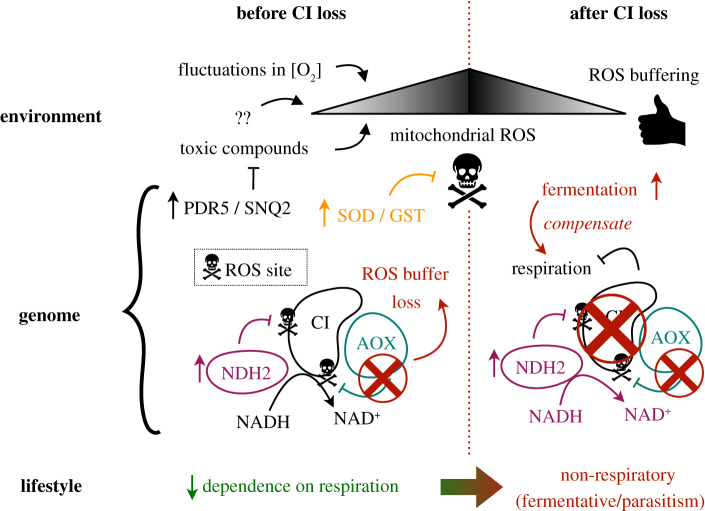
Proposed model for the causes and consequences of complex I. Fluctuations in oxygen concentrations or exposure to toxic compounds trigger the formation of mitochondrial reactive oxygen species (ROS) at CI, and organisms lacking AOX should be particularly sensitive to these (because AOX buffers ROS formation at CI). CI loss may be necessary to eliminate this major source of ROS, which is possible due to ancestrally increased alternative NADH dehydrogenase (NDH2) activity. Upon CI loss, the decreased respiration may be compensated through higher fermentation rates. Increased and/or novel function of multidrug transporters (PDR5 and SNQ2) and mitochondrial antioxidants (SOD and GST) reflect this adaptation to toxic compounds and mitochondrial ROS, respectively. Furthermore, we hypothesize that organisms that lost CI had an intrinsically lesser reliance on respiration, which is enlarged when CI is lost.

Based on the findings described here, and those of others [10,11], we propose a new hypothetical model that explains the causes and consequences of CI loss in fungi. We call this model the fermentation + oxidative stress (FOS) model (figure 5). The first assumption of this model is that CI loss occurred upon adoption of anaerobic or microaerophilic lifestyles, where producing CI can be costly [61]. Metabolic reliance in fermentation and (compensatory) duplications of alternative NDH2 may all reduce the selective benefit of CI retention. We call this assumption of the model the ‘*fermentation-first’* hypothesis, which is compatible with previously proposed ideas for the loss of CI in yeasts [10,11]. Accordingly, we predict increased fermentative capabilities and duplicated NDH2 in cases of recent CI loss. In addition, this explains why the retargeting of NDH2 into mitochondria rescues a (otherwise lethal) CI defect [56].

The second assumption of our model is novel, and necessary to explain our observed relationships between CI loss and the ancestral loss of AOX, the increased or novel mitochondrial antioxidants, and a possible exposure to toxic compounds similar to antifungal drugs. These diverse observations can be jointly explained by the interaction between CI, AOX and mitochondrial ROS [48,49]. CI has two sites of superoxide (the main source of ROS) production: the flavin group of flavin mononucleotide (FMN) and the ubiquinone (Q)-binding site [62]. Reoxygenation after anaerobic conditions leads to ROS formation in CI, in a way that can be compensated by AOX and NDH2 [11,63] in each of these sites. Losing AOX (which precedes CI loss) may reduce this ‘buffering’, in a way that CI loss could be essential for avoiding excessive oxidative damage under such conditions. This explains why facultative anaerobes benefit from CI loss, as previously proposed [10]. It is consistent with the predicted increased/novel SOD and GSTs, which may provide additional detoxification. In general, we (and others [11]) predict an electron transport chain (ETC) that lacks AOX to be more sensitive to any trigger of mitochondrial ROS. Accordingly, CI loss may be compensatory through avoidance of CI ROS and (maybe) diminished ETC (and thus CIII-derived ROS) activity. Our results suggest exposure to compounds similar to antifungal drugs in all recent CI loss. Antifungal compounds can trigger mitochondrial ROS production through the induction of respiration [51–53]. We thus propose these compounds to be a factor facilitating CI loss. Under this assumption, which we call ‘*oxidative stress-first’* hypothesis, the rewiring of fermentation is related to the decreased respiration resulting from CI loss. We consider that increased fermentation could be both an adaptation (consequence) and/or a predisposing factor (cause) of this decreased respiration, which cannot be resolved from our data. Remarkably, we note that the described evidence for the ‘*fermentation-first*’ hypothesis also supports the ‘*oxidative-stress first*’ hypothesis, but not the other way around, which indicates that a model that considers both assumptions (the FOS model proposed herein) is best supported by our results. We note that the selective pressure for maintaining increased antioxidants or drug transporters may disappear with time (i.e. when the toxic compound exposure is no longer present) or even upon CI loss (which should reduce the generated oxidative stress). This may explain the lack of apparent connection to oxidative stress in ancestral CI loss.

In sum, our scenario entails three conditions acting synergistically to favour a loss of CI: (i) pre-existence of biochemical capabilities that enabled better response to mitochondrial oxidative stress, (ii) common exposure to toxic compounds that induce mitochondrial ROS production and (iii) environments involving periods of hypoxia and return to oxygenation. Ultimately, CI loss locked these species in a lifestyle less dependent on OXPHOS-dependent energy, which resulted in further changes in their metabolism and transport systems. Our observations of some changes in fermentative enzymes having occurred before the loss of CI suggest that a fermentative lifestyle probably pre-dated the loss. However, our results suggest that it is oxidative stress, and not fermentation, which more directly relates to CI loss. This implies that there could be other ways, unrelated to a fermentative lifestyle, to reach CI loss preconditions similar to the three mentioned above. In this way, the oxidative-first hypothesis but not the fermentation-first hypothesis could be extrapolable to other cases of CI loss in non-fermentative species such as European mistletoe [14,34] or *Perkinsela* [15]. They are all parasites or symbionts that obtain nutrients from their hosts and may be exposed to toxic compounds and/or hypoxia/reoxygenation periods, which would benefit from lesser mitochondrial ROS production. However, we note that these non-fungal CI− organisms can possess active AOX enzymes [34,36], which means that the loss of AOX as a driver of CI loss may be exclusive of fungi. However, we consider that the taxon sampling around non-fungal CI losses is insufficient for robust comparative genomics studies. Incidentally, several anaerobic eukaryotes (such as *Blastocystis* [64]) adapted to low oxygen environments by losing CIII and CIV while retaining CI, which suggest that alternative constraints might be acting in these species. Importantly, the anaerobic stramenopile *Blastocystis* retained AOX, further indicating that AOX loss is not necessarily a proxy for adaptation to anaerobic lifestyles (which was proposed by prior models for CI loss in fungi [10]).

Interestingly, our model predicts that an organism that was able to lose CI (which is probably not equivalent to isolated deletion) is more resilient to mitochondrial ROS formation. We thus hypothesize that, regardless of the trigger of CI loss, CI− species should be more resistant to fluctuations in environmental oxygen, antifungal drugs and/or any other inductor of such oxidative stress. This may explain why the CI− yeast *C. glabrata* (the second most prevalent cause of candidiasis [65]) has intrinsic lower susceptibility to azoles (compared to other pathogenic *Candida* species) [66,67] and frequent adaptation to echinocandins [68,69], which hampers efficient treatment. This hypothesis opens the door for novel therapeutic approaches to these infections, which may rely on the induction of respiration to compensate for their inherent lower production of ROS. Importantly, this model is consistent with a previously proposed hypothesis, which states that the close relative *S. cerevisiae* has a particular metabolism (including the lack of CI) that generates less ROS [70].

Our hypothesis relies on a rather small number of identified events of recent CI loss, and therefore has a limited statistical significance. Another limitation of our study is that our inferences are based on homology relationships, which do not always equate to confirmed biochemical activities. However, we consider that the level of observed convergence is striking and reveals functional trends that could explain other cases of CI loss in fungi. Our model could be reinforced by the finding of consistent trends in additional cases of recent CI loss, or by empirical testing of some of our predictions. Our model is probably not directly extrapolable to cases of CI loss in non-fungal organisms that exhibit different metabolic properties or lifestyles, although some of its aspects may be. Of note, it is important to stress that there might be alternative evolutionary pathways and constraints that lead to CI loss in different organisms and, conversely, that the triggering factors identified here may not inevitably lead to CI loss, as alternative adaptive mechanisms might exist. Finally, it remains unclear whether the higher number of CI loss events found in fungi as compared to other eukaryotic groups reflect some intrinsically higher propensity or rather existing biases in genomic sampling.

## Data accessibility

The data underlying this article are available in the Sequence Read Archive with the accession code PRJNA663430 and can be accessed at http://www.ncbi.nlm.nih.gov/bioproject/663430.
